# Biomarkers of systemic lupus erythematosus identified using mass spectrometry‐based proteomics: a systematic review

**DOI:** 10.1111/jcmm.13031

**Published:** 2016-11-23

**Authors:** Orthodoxia Nicolaou, Andreas Kousios, Andreas Hadjisavvas, Bernard Lauwerys, Kleitos Sokratous, Kyriacos Kyriacou

**Affiliations:** ^1^ Department of Electron Microscopy/Molecular PathologyThe Cyprus Institute of Neurology and GeneticsNicosiaCyprus; ^2^ Department of Electron Microscopy/Molecular PathologyCyprus School of Molecular MedicineNicosiaCyprus; ^3^ Department of RheumatologyUniversité catholique de LouvainBruxellesBelgium

**Keywords:** biomarkers, systemic lupus erythematosus, SLE, mass spectrometry, proteomics, systematic review

## Abstract

Advances in mass spectrometry technologies have created new opportunities for discovering novel protein biomarkers in systemic lupus erythematosus (SLE). We performed a systematic review of published reports on proteomic biomarkers identified in SLE patients using mass spectrometry‐based proteomics and highlight their potential disease association and clinical utility. Two electronic databases, MEDLINE and EMBASE, were systematically searched up to July 2015. The methodological quality of studies included in the review was performed according to Preferred Reporting Items for Systematic Reviews and Meta‐analyses guidelines. Twenty‐five studies were included in the review, identifying 241 SLE candidate proteomic biomarkers related to various aspects of the disease including disease diagnosis and activity or pinpointing specific organ involvement. Furthermore, 13 of the 25 studies validated their results for a selected number of biomarkers in an independent cohort, resulting in the validation of 28 candidate biomarkers. It is noteworthy that 11 candidate biomarkers were identified in more than one study. A significant number of potential proteomic biomarkers that are related to a number of aspects of SLE have been identified using mass spectrometry proteomic approaches. However, further studies are required to assess the utility of these biomarkers in routine clinical practice.

## Introduction

SLE is a chronic autoimmune inflammatory disease with a broad spectrum of clinical manifestations, affecting the majority of organs and tissues. In most cases, vital organs are involved including brain, heart, joints, skin and kidneys 1. Remarkably, the disease occurs more often in women with a female‐to‐male ratio of 9:1 and has a higher incidence in non‐Caucasian populations 2. SLE is characterized by the presence of high titres of autoantibodies directed against a broad range of self‐nuclear antigens. Accumulation of SLE autoantibodies in the host tissues and formation of immune complexes, activate production of immune system cells that perpetuate a positive feedback loop resulting in organ damage 3. Despite intensive research, the precise aetiology and pathogenic mechanisms underlying SLE are poorly understood. It is believed that SLE results from the interaction between genetic, epigenetic, environmental, hormonal and immunoregulatory factors 1.

The diagnosis of SLE is challenging due to its heterogeneous nature, variable clinical presentation and unpredictable course with periods of remission and flares 1. Currently, patients need to fulfil at least 4 of the 11 clinical and laboratory criteria outlined by the American College of Rheumatology (ACR), for the formal diagnosis of SLE 4, 5. However, these criteria were formulated and validated for the classification of patients with established disease and might exclude patients with early signs or limited disease. Evidence from tertiary care centres suggests that only 60% of the patients with SLE meet the ACR criteria 6.

Besides the pressing need to improve the classification of patients with SLE, assessment of disease activity remains another important aspect in the management of patients with SLE. At present, conventional disease assessment methods, including the use of acute phase markers and autoimmune serologic tests (*e.g*. anti‐double‐stranded DNA antibodies), are of limited sensitivity and specificity. In lupus nephritis, renal biopsy remains the ‘gold standard’ not only for assessing disease activity, but also for assessing prognosis and monitoring therapy 7. However, this is an invasive and cumbersome procedure that causes discomfort to the patients. Therefore, there is an urgent need for discovering reliable SLE biomarkers that can be used not only for diagnosis, but also for disease classification, monitoring, identification of organ involvement and better prediction of response to therapy.

A proteomic biomarker is defined as ‘a specific peptide/protein that is associated with a specific condition, such as the onset, the manifestation, or progression of a disease or a response to treatment’ 8. During the last two decades, advances in mass spectrometry (MS) enable the identification and quantification of thousands of proteins in complex biological samples, in a single run 9. Application of MS‐based proteomics to SLE provides unprecedented opportunities for identifying novel protein biomarkers, which can be used for early diagnosis and contribute to a more effective patient management. The aim of the current systematic review is to summarize and evaluate protein biomarkers identified in patients with SLE, in different biological specimens using MS. In addition, our objective was to provide more comprehensive information about the number and relevant biological function of the proteomic biomarkers detected in SLE, as well as their possible diagnostic and therapeutic utility. This systematic review also outlines the challenges that need to be addressed in future research endeavour related to the discovery of SLE proteomic biomarkers.

## Materials and methods

### Data sources and searches

We performed a systematic review of the literature on the discovery of proteomic biomarkers in patients with SLE using MS‐based proteomic approaches. Relevant studies were identified by searching two electronic databases, MEDLINE and EMBASE, in July 2015. The search strategy in MEDLINE (1950–July 2015) was developed using the following search terms: [(‘Lupus Erythematosus, Systemic’[Mesh] OR ‘Systemic Lupus Erythematosus’ OR ‘SLE’ OR ‘Lupus nephritis’ OR ‘Lupus’) AND (‘Mass Spectrometry’[Mesh] OR ‘Mass Spectrometry’ OR ‘Electrophoresis, Gel, Two‐Dimensional’[Mesh] OR ‘Two‐Dimensional Electrophoresis’ OR ‘proteome’ OR ‘proteomics’)]. The literature search in EMBASE (1988–July 2015) was conducted in a similar way using the terms: [‘Systemic Lupus Erythematosus’ OR ‘SLE’ OR ‘Lupus nephritis’ OR ‘Lupus’) AND (‘Mass Spectrometry’ OR ‘Two‐Dimensional Electrophoresis’ OR ‘proteome’ OR ‘proteomics’)]. Searching results from the two databases were imported in the bibliographic management software Endnote X5 (Thomson Reuters, PA, USA). Duplicates were automatically removed. The articles were reviewed in a two‐stage procedure. In the first stage of the review process, abstracts of all identified articles were screened. Review articles, editorials, case reports, letters to the editor, conference abstracts, notes and news were excluded. In the second stage of the review process, full texts of the remaining studies were evaluated. A checklist of specified inclusion criteria was used to ensure uniformity in the assessment of the identified manuscripts. The final articles that were selected all fulfilled the following eligibility criteria: written in English, used human samples, compared biological fluids from patients with SLE with a control group (healthy or other type of control group), referred to SLE, were informative about the type of biological fluid used, provided details about the mass spectrometry technique used as well as the proteomic biomarkers identified. Articles that did not meet one or more of these inclusion criteria were excluded. This review was performed by two independent reviewers (ON and AK) based on Preferred Reporting Items for Systematic Reviews and Meta‐analyses (PRISMA) guidelines 10. In case of any unsolved discrepancies between the two reviewers, a third reviewer (KS) was consulted.

## Results

The initial literature search identified 1093 records. After duplicate removal, 775 records remained. These were all screened, resulting in a total of 25 selected full‐text articles, published between 2007 and 2015, which were included in this systematic review. The detailed review process with the eligibility criteria used is shown in Figure S1. The main findings of each article are summarized in Table 1. The table is divided into two parts. The first part includes targeted and untargeted proteomic studies, whereas the second part lists the validation studies. Information about specimen type, controls, proteomic/validation techniques used, biomarkers identified, disease association and/or possible clinical use of the biomarkers is also included. Additionally, the 25 articles were assessed according to the recommendations proposed for biomarker identification and qualification in clinical proteomics (Table S1) 8. All selected articles fulfilled at least four of the eight recommendations. It is noted that only the first criterion, ‘justification and description of clinical question, outcome and selection of subjects’, is fulfilled by all the selected articles.

**Table 1:** Summary of the main findings of the reviewed articles

Reference	Specimen type	Initial phase targeted and untargeted proteomics			Validation phase			Disease association and/or possible Clinical Use
		Sample size (SLE/Control)	Proteomic techniques (confirmation techniques)	Total number of identified proteins /number of differentially expressed proteins	Specimen type/Sample size	Validation techniques	Biomarker/Protein signature	
Alaiya *et al*. 32	Renal biopsies	6 LN class IV‐Global, 5 LN class IV‐Segmental/3 ANCA‐GN, 3 normal kidneys	2‐DE, MALDI‐TOF MS	28 proteins in total /9 proteins only if subgroups IV‐G and IV‐S were classified as one group	Renal biopsies /5 LN class IV‐G /4 ANCA, 3 normal kidneys	Label‐free LC/MS/MS	Albumin, annexin A5, cytokeratin 18, cytokeratin 19, serotransferrin	LN diagnosis (class IV) molecular subclassification of LN; Subcategories of class IV to IV‐G and IV‐S not established
Caster *et al*. 11	Serum	10 PLN, 15 MLN /10 non‐LN SLE	SDS‐PAGE, LC‐MS/MS (Sera immunoblotted against cultured podocyte's membrane and human glomerular extracts)	102 total common proteins in glomerular and podocytes extracts/36 of those proteins were membrane‐associated	Serum /10 PLN, 10 MLN /10 non‐LN SLE, 10 HC	ELISA analysis	Elevated anti‐annexin A2 in PLN patients compared with MLN, non‐LN SLE and HC with a *P* value < 0.01^a^	Diagnosis of proliferative forms of LN
Dai *et al*. 19	PBMCs	9 Female SLE^b^ /7 age‐matched HC	2‐DE, MALDI‐TOF/TOF MS	5 Proteins total; 4 upregulated 1 downregulated	N/A	N/A	N/A	SLE Diagnosis
Fang *et al*. 35	Skin biopsies	10 SLE (affected, untreated skin) /10 HC (normal skin)	2‐DE, MALDI‐TOF MS WB (Conf.) IHC (Conf.)	18 proteins total /Focused on keratins; 2 downregulated 6 upregulated	N/A	N/A	N/A	SLE skin lesions
Huang *et al*. 12	Serum	32 SLE/43 DC^d^ 43 age‐ and sex‐matched HC	MALDI‐TOF MS combined with WCX magnetic beads	60 protein peaks in total; 32 downregulated 28 upregulated /Classification tree analysis resulted in 4 protein peaks: 2 downregulated 2 upregulated	Serum /32 SLE /42 DC, 40 age‐ and sex‐matched HC	Blinded testing set (WCX magnetic beads, MALDI‐TOF MS)	Panel of proteins with *m/z* ratio 4070.09, 7770.45, 28 045.1, 3376.02 differentiate samples of SLE from other autoimmune diseases and HC with accuracy of 78.1, 85.8 and 90%, respectively	SLE diagnosis
Iizuka *et al*. 13	Serum	30 CNS‐SLE (CSF from 3 patients) /30 non‐CNS‐SLE, 5 HC	2‐DE, MALDI‐TOF/TOF MS	4 differentially expressed proteins (autoantigens)	N/A	N/A	N/A	CNS lupus diagnosis
Kazemipour *et al*. 14	Serum	13 SLE /7 HC	2‐DE, MALDI‐TOF/TOF MS	9 differentially expressed proteins; 3 upregulated 6 downregulated	N/A	N/A	N/A	SLE diagnosis
Kimura *et al*. 15	Serum	7 NPSLE/12 HC	2‐DE, WB, LC‐MS/MS (using rat brain as antigen source)	6 proteins (antigens) in total / only Rab guanosine diphosphate dissociation inhibitor α (αGDI) was brain‐specific antigen and located in neurons	Serum /18 NPSLE /19 SLE without NP symptoms, 45 DC^e^, 12 HC	1‐DE WB against human anti‐aGDI full‐length recombinant protein	Anti‐aGDI was higher in NPSLE patients with psychosis (80%) compared with NPSLE without psychosis	Diagnosis of psychosis in NPSLE
Li *et al*. 20	CD4^+^ T cells from PBMCs	10 Female SLE^b^ / unknown number of age‐ and sex‐matched HC	SDS‐PAGE, LC‐MS/MS (CD4^+^ T cells immunoprecipitated with anti‐human Gadd45a antibody WB (Conf.) RT‐PCR (Conf.)	30 proteins that bind to Gadd45a in total /focused on, high‐mobility group box protein 1 (HMGB1); (upregulated)	N/A	N/A	N/A	SLE disease activity (positive correlation with SLEDAI) SLE pathogenesis (HMGB1 protein binds to Gadd45a contributing to DNA demethylation in CD4^+^ T cells)
Morgan *et al*. 16	Serum	7 SLE /7 age‐, sex‐ and race‐matched HC	2‐DE, LC‐MS/MS	4 downregulated proteins	Serum /11 LN, 24 non‐LN SLE /8 HC	ELISA	Apolipoprotein CIII increased in LN compared with non‐LN SLE and HC	LN diagnosis Increased atherosclerotic risk in LN
Mosley *et al*. 29	Urine	26 SLE with active LN /49 SLE with inactive LN, 15 HC	SELDI‐TOF MS	32 protein ions in total / 2 protein ions best discriminate the two groups with 92% specificity and 92% sensitivity *P* value < 0.01^a^	Sequential urines /6 SLE with biopsies indicative for active disease	SELDI‐TOF MS	*m/z* 3340 and 3980 predicted a change in disease state prior to clinical classification	LN activity diagnosis LN relapse and remission prediction
Nielsen *et al* 25	Platelet‐poor plasma	12 SLE^c^ /12 DC^f^,12 HC	LC‐MS/MS Label‐free, spectral count Flow cytometry (Conf.)	Increased MP‐associated IgG, IgM and C1q in patients with SLE (highest levels in active disease)	N/A	N/A	N/A	SLE disease activity and pathogenesis
Nielsen *et al*. 26	Platelet‐poor plasma	44 SLE /36 age‐ and sex‐matched HC	LC‐MS/MS Label‐free, peptide intensities Flow cytometry (Conf.)	Increased MP‐associated galectin‐3‐binding protein (G3BP), C1q and Ig in patients with SLE	Renal biopsies /3 SLE with LN /1 HC	IEM	Anti‐G3BP and anti‐IgG colocalized in the glomeruli in all 3 biopsies	LN pathogenesis (no association with disease activity or clinical manifestations)
Ostergaard *et al*. 27	Platelet‐poor plasma	12 SLE /12 DC^g^, 12 HC	LC‐MS/MS Label‐free, peptide intensities	531 MP‐associated proteins in total /248 proteins; 191 upregulated 57 downregulated	N/A	N/A	N/A	SLE diagnosis Disease activity
Pavon *et al*. 21	PBMCs	6 SLE /8 age‐matched HC	2‐DE, MALDI‐TOF MS MALDI‐TOF/TOF MS (Conf.)	98 proteins total /ID two S100A9 protein isoforms and their phosphorylated counterparts; S100A9‐S, S100A9‐1 (phosphorylated at Thr^113^)	PBMCs /30 SLE /30 HC 18 new SLE subjects/ 9 new HC	WB	S100A9 increased HSP 90α/β, HSP 70 and PPIase A decreased	S100A9 proteomic signature for the abnormal presence of activated low‐density granulocytes in SLE PBMCs
					PBMCs / 14 SLE	RT‐PCR	S100A9 mRNA increased	
Serada *et al*. 17	Serum	41 SLE (23 LN) /60 DC^h^, 19 HC	2D‐PAGE, WB, LC‐MS/MS Sera immunoblotted with human umbilical vein endothelial cells ELISA (Conf.)	6 proteins total / one novel autoantigen; Aldolase A	N/A	N/A	N/A	LN diagnosis
Somparn *et al*. 30	Urine	5 active LN / 5 age‐ and sex‐matched inactive LN	2‐DE, ESI‐Q‐TOF MS/MS	14 proteins in total	Urine /30 active LN /26 inactive, 14 non‐LN glomerular diseases, 8 age‐ and sex‐matched HC	ELISA to validate differential levels of Prostaglandin H_2_D‐isomerase and Z_n_‐α2‐glycoprotein	Prostaglandin H_2_D‐isomerase increased in active LN compared with the other 3 groups	LN activity diagnosis (class III or IV)
Sui *et al*. 33	Renal biopsies	10 Female LN b(3 class V and 7 not reported) /3 age‐ and sex‐matched HC	LC‐MS/MS iTRAQ‐labelled	512 proteins in total /18 proteins with difference of more than 1.5‐fold; 9 upregulated proteins 7 downregulated Proteins (2 unnamed proteins)	N/A	N/A	N/A	LN diagnosis
Sun *et al*. 36	CSF	27 NPSLE before and 2 weeks after treatment /17 scoliosis, 10 SLE without NP manifestations	MALDI‐TOF MS combined with WCX magnetic beads	12 protein peaks total; 10 upregulated and 2 downregulated /Decision tree recognizes NPSLE with 92.6% sensitivity and specificity: Panel m/z peaks 8595, 7170, 7661, 7740, 5806 (3 upregulated and 2 downregulated)	CSF /Blind test group: 12 NPSLE before treatment /12 lumbar disc herniation, 9 with other autoimmune diseases with NP involvement CSF /1 NPSLE incubated with anti‐ubiquitin polyclonal Ab for 2 hr /same CSF sample incubated with normal rabbit polyclonal Ab (negative control) CSF /16 NPSLE /7 SLE without NP manifestations	Immunoprecipitation, MALDI‐TOF MS combined with WCX magnetic beads Immunoprecipitation, MALDI‐TOF MS combined with WCX magnetic beads WB (Conf.) ELISA	Decision tree recognizes NPSLE with 91.7% sensitivity and 85.7% specificity: Panel of m/z peaks 8595, 7170, 7661, 7740, 5806 8595 peak identified as ubiquitin, downregulated in CSF of NPSLE after treatment Anti‐ubiquitin levels higher in NPSLE group	NPSLE diagnostic proteomic model (Panel of m/z peaks) Disease activity (CSF ubiquitin levels)
Wang *et al*. 22	PBMC	6 active SLE /6 stable SLE, 6 age‐ and sex‐matched HC	MALDI‐TOF/TOF MS iTRAQ‐labelled	Unknown number of total proteins/focused on STRAP downregulated in active SLE	PBMC /14 active SLE /11 stable SLE, 11 HC	WB	Lower expression of STRAP in active SLE	SLE activity (STRAP is inversely correlated with *SLEDAI*)
Wang *et al*. 23	PBMC	6 active SLE, 6 stable SLE /6 rheumatoid arthritis (RA), 6 age‐ and sex‐matched HC	MALDI‐TOF/TOF MS iTRAQ‐labelled	452 proteins in total /67 differentially expressed unique proteins;	N/A	N/A	N/A	SLE diagnosis SLE disease activity Discrimination of SLE from RA
Wu *et al*. 40	Serum	7 SLE family cases /63 individual SLE, 83 HC	MALDI‐TOF MS combined with WCX magnetic beads	4 discriminative protein peaks; 1 upregulated protein peak 3 downregulated protein peaks	N/A	N/A	N/A	SLE risk prediction
Zhang *et al*. 31	Urine	19 Female LN^b^ who experienced 25 flares (5 class III /11 class IV /3 class V) Samples taken: pre‐flare, flare, treatment and baseline	SELDI‐TOF MS, Direct on‐chip peptide sequencing LC‐MS/MS (Conf.)	176 protein peaks in total; 27 differentially expressed protein ions between flare intervals; / selected peaks resulted in identification of 2 proteins;	Renal biopsies /3 LN class IV /1 HC	IHC (staining with anti‐hepcidin polyclonal Ab)	Increased expression of hepcidin in SLE nephritis patients compared with controls	SLE renal flare prediction
Zhou *et al*. 24	PBMCs	14 Female SLE^b^ /9 age‐ and sex‐matched HC	2‐DE, LC‐MS/MS	16 proteins in total; 5 upregulated 11 downregulated /Focused on annexin A5	PBMCs /47 Female SLE^b^ /31 age‐ and sex‐matched HC	WB	Upregulation of annexin A5 in SLE	SLE‐related thrombophilia (increased intracellular and decreased serum annexin A5 levels are protective from lupus‐related thrombophilia)
					Serum /123 Female SLE^b^ /113 age‐ and sex‐matched HC	ELISA	Downregulation of annexin A5 in SLE sera Anti‐annexin A5 levels did not greatly differ between SLE and controls	
					Platelet‐poor plasma /30 Female SLE^b^ /30 age‐ and sex‐matched HC	Coagulation assays	Elevated annexin A5 could shorten prothrombin time, activated partial thromboplastin time, prolonged thrombin time	
Zhou *et al*. 18	Serum	Group 1:12 SLE Females^b^ with LN and FH Group 2:12 SLE Females^b^ with LN but no FH Group 3:12 SLE Females^b^ without LN or FH/ Controls: 6 sex‐matched HC	MALDI‐TOF MS combined with WCX magnetic beads	6 protein peaks in total / 1 peak upregulated in groups 1,2 and 3, 1 peak present only in groups 1,2 and 3 3 peaks present only in groups 1 and 2 1 peak present only in group 1	N/A	N/A	N/A	SLE diagnosis SLE genetic basis LN diagnosis

LN: lupus nephritis; ANCA: antineutrophil cytoplasmic antibody vasculitis; GN: glomerulonephritis; 2‐DE: two‐dimensional gel electrophoresis; MALDI: matrix‐assisted laser desorption/ionization; TOF: time‐of‐flight mass spectrometry; MS: mass spectrometry; PLN: proliferative LN; MLN: membranous LN; SDS‐PAGE: sodium dodecyl sulphate–polyacrylamide gel electrophoresis; LC‐MS: liquid chromatography MS; HC: healthy controls; ELISA: enzyme‐linked immunosorbent assay; PBMCs: peripheral blood mononuclear cells; N/A: not applicable: without validation; SLE: systemic lupus erythematosus; Conf.: confirmation techniques; WB: Western blot analysis; IHC: immunohistochemistry; DC: disease controls; WCX: weak cation exchange chromatography; CNS: central nervous system; CSF: cerebrospinal fluid; NPSLE: neuropsychiatric SLE; Gadd45a: growth arrest and DNA damage‐inducible‐alpha; SLEDAI: SLE disease activity index; SELDI: surface‐enhanced laser desorption/ionization; MP: microparticle; IEM: immune electron microscopy; ID: identified; S100A9: S100 calcium‐binding protein A9; HSP 90α/β: heat‐shock protein 90α/β; HSP 70: Heat‐shock protein 70; PPIase A: peptidyl‐prolyl cis‐trans isomerase A; RT‐PCR: reverse transcription–polymerase chain reaction; iTRAQ: isobaric tagging reagent for absolute quantitation; 2D‐PAGE: two‐dimensional polyacrylamide gel electrophoresis; ESI‐Q‐TOF MS/MS: electrospray ionization quadrupole time‐of‐flight mass spectrometry; Ab: antibody; STRAP: serine‐threonine kinase receptor‐associated protein.

a *P* value stated only if other than 0.05.

b Gender stated only if study did not use both genders.

c Twelve patients with SLE were divided into four groups of three patients each: Group I: biopsy‐proven active nephritis and high disease activity (SLEDAI > 12), Group II: biopsy‐proven active nephritis, current quiescent disease (low SLEDAI = 0), Group III: No history of nephritis, current active disease other than nephritis (high SLEDAI > 10).

d State the disease controls categories from this article: 15 rheumatoid arthritis, 15 Sjögren's syndrome, 13 systemic sclerosis.

e State the disease controls categories from this article: 12 multiple sclerosis, 13 infectious meningoencephalitis, 10 polyneuropathy, 10 psychotic syndromes.

f State the disease controls categories from this article: 6 rheumatoid arthritis, 6 systemic sclerosis.

g State the disease controls categories from this article: 6 rheumatoid arthritis, 6 systemic sclerosis.

h State the disease controls categories from this article: 49 rheumatoid arthritis, 11 polymyositis.

### Types of specimens

In the 25 selected articles, a variety of specimen types obtained from patients with SLE was used. This can be attributed to the clinical heterogeneity of the disease, affecting almost all organs and tissues. Serum was the most frequently used biological fluid (9 of the 25 studies) 11, 12, 13, 14, 15, 16, 17, 18, followed by the peripheral blood mononuclear cells (PBMCs) (6 of the 25 studies) 19, 20, 21, 22, 23, 24, and platelet‐poor plasma (3 of 25 studies) 25, 26, 27. Theoretically, blood serum or plasma may be ideal to analyse as they contain specific biomarkers for almost all human diseases, but it is recognized that potential biomarkers in these samples may be present at very low concentrations 28. Three studies 29, 30, 31 examined the urine proteome and two studies 32, 33 analysed the protein extract from kidney biopsies. Potentially, urinary biomarkers may be more meaningful as they reflect more accurately renal disease than their serum counterparts. Indeed, an emerging concept is that urine is potentially a liquid biopsy of the kidney 34. Nephritis is a common and serious complication of SLE; thus, differentially expressed proteins during renal flare might be potential novel and predictive lupus nephritis (LN) biomarkers. Another study examined the proteome from skin biopsies, as skin is the second most commonly affected organ in SLE after joint involvement 35. Finally, one study analysed cerebrospinal fluid as all parts of the nervous system can be affected in patients with SLE causing neuropsychiatric syndromes 36.

### Proteomic techniques

The term proteomics describes the large‐scale characterization of the whole protein content of a cell, organ or organism at a given time 37. Herein, the proteomic studies were classified into two broad categories. The first category includes discovery or unbiased approaches and the second targeted or biased approaches. A typical MS‐based workflow involves two main steps: (*i*) separation of proteins and peptides present in a complex biological sample using gel‐based or gel‐free techniques, (*ii*) MS‐based protein identification 38, which involves protein digestion prior to analysis 39. Alternatively, studies using targeted proteomic approaches aim to detect specific proteins, for example autoantigens. The experimental workflow is also different compared with the untargeted approach. In studies included in this systematic review, in which targeted approaches were used, the tissue of interest was separated by either one‐dimensional gel electrophoresis (1‐DE) or 2‐DE, followed by immunoblotting with antisera of patients with SLE. The immune‐reactive bands were then subjected to MS analysis for identification.

In the current systematic review, 10 studies used 2‐DE, two studies used sodium dodecyl sulphate–polyacrylamide gel electrophoresis (SDS‐PAGE) 11, 20 and one study used 2D‐PAGE combined with Western blot 17 for protein separation. Moreover, four studies 12, 18, 36, 40 used weak cation exchange (WCX) magnetic beads to enrich low molecular weight peptides prior to MS analysis. Indeed, the combination of magnetic beads with matrix‐assisted laser desorption/ionization–time‐of‐flight mass spectrometry (MALDI‐TOF MS) enables robust, precise and rapid protein profiling of complex samples 41, 42. Subsequently, protein spots were analysed by MS and proteins were identified by database searches.

A variety of MS approaches was used to obtain proteomic data from different biological samples. These include MALDI‐TOF MS, MALDI‐TOF/TOF MS, surface‐enhanced laser desorption/ionization (SELDI)‐TOF MS and liquid chromatography (LC) combined with tandem MS (LC‐MS/MS, including the use of triple‐quadrupole MS instruments and hybrid quadrupole–TOF instruments; Table 1). Both label‐free and labelled approaches were used. Particularly, 3 of the 25 studies used isobaric tags for relative and absolute quantitation, namely isobaric tagging reagent for absolute quantitation (iTRAQ)‐labelled approach 22, 23, 33, whereas 3 of 25 used a label‐free approach 25, 26, 27. Significantly, 13 of these studies validated the identified candidate biomarkers. For validation, most studies used well‐established immune‐based methods including Western blot (WB), enzyme‐linked immunosorbent assay (ELISA) and immunohistochemistry (IHC) and four studies used additional proteomic methods.

### Identified proteins

A total of 241 candidate biomarkers were identified in the 25 studies included in this review (Table 2). In 13 of the 25 studies, validation studies of a selected number of biomarkers were performed in an independent cohort, which resulted in the validation of 28 candidate biomarkers. These include albumin, annexin A5, cytokeratin 18, cytokeratin 19, serotransferrin 32, annexin A2 antibody 11, a panel of proteins with *m/z* ratio of 4070.09, 7770.45, 28045.1, 3376.02 12, Rab guanosine diphosphate dissociation inhibitor α (αGDI) antibody 15, apolipoprotein CIII 16, peaks of *m/z* 3340, m/z 3980 29, galectin‐3‐binding protein (G3BP) antibodies, anti‐immunoglobulin G (IgG) 26, S100 calcium‐binding protein A9 (S100A9), heat‐shock protein (HSP) 90 a/β, HSP70, peptidyl‐prolyl cis‐trans isomerase A (PPIase A) 21, prostaglandin H_2_D‐isomerase 30, panel of proteins with *m/z* ratio 8595 (ubiquitin), 7170, 7661, 7740, 5806 36, serine‐threonine kinase receptor‐associated protein (STRAP) 22, hepcidin 31 and annexin A5 24.

**Table 2:** Summary of identified biomarkers in different specimen types

Specimen type	Disease association	Biomarkers	Reference
Serum	SLE	***m/z*** **ratio 4070.09, 7770.45, 28045.1, 3376.02**; Apolipoprotein A‐I, prothrombin, keratin type II (cytoskeletal), keratin 1, albumin, type II keratin, transthyretin, haptoglobin 2; *m/z* ratio 9342.23, 4094.03, 5905.35, 7973.53	Huang *et al*. 12 Kazemipour *et al*. 14 Wu *et al*. 40
	LN	**Annexin A2 antibody**, ATP synthase subunit‐alpha, mitochondrial**,** ATP synthase subunit‐beta, mitochondrial, alpha‐enolase ENO1, Moesin, glyceraldehyde‐3‐phosphate dehydrogenase, Elongation factor 1‐alpha 1, guanine nucleotide‐binding protein G(i) subunit‐alpha‐2, isoform 2 of AP‐2 complex subunit mu, isoform 2 of protein disulphide isomerase A6, pyruvate kinase PKM, 60‐kD heat‐shock protein mitochondrial, actin‐related protein 3, V‐type proton ATPase subunit B brain isoform, myosin‐9, isoform 3 of heterogeneous nuclear ribonucleoprotein, septin‐7, isoform 2 of coronin‐1C, tubulin‐beta‐4A chain, T‐complex protein 1 subunit‐gamma, isoform 2 of ATP‐dependent RNA helicase, isoform 2 of basigin, dolichyl‐diphosphooligosaccharide–protein glycosyltransferase 48‐kD subunit, heat‐shock protein HSP 90‐beta, ezrin, isoform 2 of neutral cholesterol ester hydrolase 1, isoform 2 of heat‐shock cognate 71‐kD protein, heterogeneous nuclear ribonucleoprotein U, Rho GTPase‐activating protein 1, isoform 2 of fatty aldehyde dehydrogenase, serine palmitoyltransferase 1, isoform 2 of ATP‐citrate synthase; IgM heavy chain, **apolipoprotein CIII**, HSA fragment, haemopexin fragment; glyceraldehyde‐3‐phosphate dehydrogenase, heterogeneous nuclear ribonucleoprotein A2/B1, annexin A2, aldolase A, elongation factor 1‐gamma, lupus La protein (SS‐B/La); m/z 4207 Da, 2658 Da, 1465 Da, 5332 Da, 5900 Da, 1943 Da	Caster *et al*. 11 Morgan *et al*. 16 Serada *et al*. 17 Zhou *et al*. 18
	CNS‐SLE	Peroxiredoxin‐4, ubiquitin carboxyl‐terminal hydrolase isozyme L1, splicing factor arginine/serine‐rich 3, histone H2A type 1	Iizuka *et al*. 13
	NPSLE	Stress‐70 protein, **Rab guanosine diphosphate dissociation inhibitor α antibody**, Isocitrate dehydrogenase [NAD] subunit‐alpha, L‐lactate dehydrogenase B chain, F‐actin‐capping protein subunit‐alpha‐2, Rab guanosine diphosphate dissociation inhibitor‐beta	Kimura *et al*. 15
PBMCs	SLE	Immunoglobulin J chain, apolipoprotein A‐IV precursor, glutathione S‐transferase, calprotectin L1H, zinc finger protein subfamily 1A; high‐mobility group box protein 1 (CD4^+^ T cells); **S100 calcium‐binding protein A9, heat‐shock protein 90α/β**,** heat‐shock protein 70**,** peptidyl‐prolyl cis‐trans isomerase A**;** serine‐threonine kinase receptor‐associated protein;** cDNA FLJ61039, AF4/FMR2 family member 1, cDNA FLJ55107, structural maintenance of chromosome protein 3, protein S100‐A9, protein S100‐A8, protein S100‐A12, lysozyme C, glutathione S‐transferase kappa 1 isoform c, isoform 2 of zinc finger protein 549, 26‐kD protein, histone H2A type 1, myeloblastin, brain acid‐soluble protein 1, protein S100‐P, neutrophil defensin 1, isoform 2 of metalloendopeptidase OMA1 (mitochondrial), 42‐kD protein, latent transforming growth factor‐beta‐binding protein 1 isoform, resistin, AF4/FMR2 family member 1, cDNA FLJ61340, HLA class I histocompatibility‐alpha chain, histone H1.2, cDNA FLJ51589, putative uncharacterized protein GCA, FCGR3B protein, nucleolar protein 5A, serine‐threonine kinase receptor‐associated protein, myosin regulatory light polypeptide 9, isoform A of bromodomain and WD repeat‐containing protein 1, vacuolar protein sorting‐associated protein 35, putative uncharacterized protein PTMA, isoform 2 of retinol dehydrogenase 11, ribosomal protein L10 (fragment), cathepsin A isoform a precursor, high‐mobility group protein B2, protein disulphide isomerase A4, 17‐kD protein, NADH dehydrogenase [ubiquinone] 1‐alpha subcomplex subunit 4, isoform 1 of multimerin‐1, p180/ribosome receptor, NUMA1 variant protein (fragment), 20‐kD protein, dihydrolipoyllysine residue succinyltransferase component of 2‐oxogluterate dehydrogenase complex (mitochondrial), isoform 2 of heterochromatin protein 1‐binding protein 3, 16‐kD protein, cDNA FLJ51702, myosin regulatory light chain MRCL2 isoform B, apolipoprotein C‐I, MHC class I antigen (fragment), protein XRP2, tubulin‐alpha‐1B chain, Ras‐related C3 botulinum toxin substrate 2, leucocyte antigen HLA‐A, 51‐kD protein, HLA class I histocompatibility antigen (B‐58‐alpha chain), isoform 2 of retinol dehydrogenase 11, ribosomal protein L10 (fragment), cDNA FLJ55509, platelet basic protein, membrane‐associated progesterone receptor component 1, hypothetical protein XP_02342881, isoform 1 of reticulon‐4, putative uncharacterized protein PARVB, isoform 1 of protein unc‐13 homologue D, putative uncharacterized protein LCN2, cathelicidin antimicrobial peptide precursor, 66‐kD protein	Dai *et al*. 19 Li *et al*. 20 Pavon *et al*. 21 Wang *et al*. 22 Wang *et al*. 23
	SLE Thrombophilia	**Annexin A5,** glyceraldehyde‐3‐phosphate dehydrogenase, integrin‐linked protein kinase, adenylylcyclase‐associated protein 1, transketolase, proline‐serine‐threonine phosphatase‐interacting protein 2, triosephosphate isomerase, tyrosine‐protein kinase CSK, dynamin‐1‐like protein, elongation factor 1‐alpha 1, T‐complex protein 1 subunit zeta, heat‐shock protein‐beta‐1, phosphoglycerate kinase 1, alpha‐enolase, osteoclast‐stimulating factor 1, heat‐shock cognate 71 kD protein	Zhou *et al*. 24
Platelet‐poor plasma	SLE	IgG‐MPs, IgM‐MPs, C1q‐MPs (proteins discussed by the author out of 248 proteins)	Nielsen *et al*. 25 Ostergaard *et al*. 27
Urine	LN	MP‐G3BP, MP‐C1q (3 subunits), MP‐Ig (most abundant: IgJ, IgM, IgG2), Ig (**IgG**, IgM, IgA), complement proteins (C1), fibronectin, 14‐3‐3^η^, desmosomal proteins, ficolin 2, **galectin‐3‐binding protein,** β_2_‐glycoprotein I, β_6_‐tubulin, β_2C_‐tubulin, lysosome‐associated membrane protein 1, transforming factor β1; ***m/z*** **ratio 3340, 3980; prostaglandin H**_**2**_ **D‐isomerase**, serotransferrin, alpha‐1‐glucoprotein, alpha‐2‐HS‐glycoprotein, haptoglobin, alpha‐1‐antitrypsin, albumin, Zn‐alpha‐2‐glycoprotein, Ig kappa chain V‐III SLE region, Ig kappa chain V‐III HAH region, Ig kappa chain C region, retinol‐binding protein 4, beta‐2‐microglobulin, transthyretin, **hepcidin** (isoforms 20 and 25)**,** a1‐antitrypsin, N‐terminal region of albumin	Nielsen *et al*. 25 Mosley *et al*. 29 Somparn *et al*. 30 Zhang *et al*. 31
Biopsies	LN	Renal: Ezrin P81, **serotransferrin**,** cytokeratin 18, cytokeratin 19**, alpha‐1‐antitrypsin, **albumin**, plasma glutathione peroxidase, 1433 protein epsilon, **annexin A5**; heterogeneous nuclear ribonucleoproteins A2/B1 isoform B1, lamin A protein, mimecan preproprotein, annexin A1, annexin A2 isoform 2, alpha‐1‐antitrypsin precursor, glutathione S‐transferase‐P1c, adenine phosphoribosyltransferase isoform a, collagen type VI alpha‐3 (isoform CRA_h), formiminotransferase cyclodeaminase form C, aldolase B, aldehyde dehydrogenase, 2‐oxoglutarate dehydrogenase (mitochondrial isoform 1 precursor), L‐arginine:glycine amidinotransferase, pyrroline‐5‐carboxylate dehydrogenase, antiquitin	Alaiya *et al*. 32 Sui *et al*. 33
	SLE Skin lesions	Skin: keratin 10, keratin 16, keratin 14, Keratin 6, keratin 5, keratin 2e, keratin 1, involucrin	Fang *et al*. 35
CSF	CNS‐SLE	m/z peaks **8595 (ubiquitin)**,** 7170, 7661, 7740, 5806**	Sun *et al*. 36

Only highlighted (bold) proteins in table were validated by the authors.

Due to the systemic, chronic and heterogeneous nature of the disease, the selected studies covered a wide range of applicability of the potential biomarkers relating to different aspects of SLE management including disease diagnosis and activity, or specific organ involvement. Interestingly, 11 potential biomarkers were identified independently in more than one study (Table 3) and further details are presented in the discussion section.

**Table 3:** Βiomarkers identified in multiple studies biomarkers in different specimen

	Biomarker	Studies	Specimen type	Sample size (SLE /Control)	Proteomic techniques	Validation	Disease association and/or possible clinical use
1	Annexin A2	Caster *et al*. 11	Serum (autoantibodies)	10 PLN/ 15 MLN, 10 non‐LN SLE	SDS‐PAGE, LC‐MS/MS	Yes	Diagnosis of proliferative forms of LN
		Serada *et al*. 17	Serum (autoantibodies)	41 SLE (23 LN)/ 60 DC, 19HC	2D‐PAGE, WB, LC‐MS/MS ELISA	No	LN Diagnosis
		Sui *et al*. 33	Renal Biopsies	10 LN(3 class V and 7 not reported)/ 3 age‐ and sex‐matched HC	LC‐MS/MS iTRAQ‐labelled	No	LN Diagnosis
2	Annexin A5	Alaiya *et al*. 32	Renal Biopsies	6 LN class IV‐Global 5 LN class IV‐Segmental /3 ANCA‐GN, 3 normal kidneys	2‐DE, MALDI‐TOF MS	Yes	LN diagnosis
	Annexin A5	Zhou *et al*. 24	PBMCs	14 SLE/ 9 age‐ and sex‐ matched HC	2‐DE, LC‐MS/MS	Yes	SLE‐related thrombophilia (increased intracellular and decreased serum annexin A5 levels are protective)
3	Alpha‐1‐antitrypsin	Somparn *et al*. 30	Urine	5 active LN/ 5 age‐ and sex‐matched inactive LN	2‐DE, ESI‐Q‐TOF MS/MS	No	LN activity and diagnosis
		Zhang *et al*. 31	Urine	19 LN (5 class III/ 11 class IV/ 3 class V). Samples taken: pre‐flare, flare, treatment and baseline	SELDI‐TOF MS, Direct on‐chip peptide sequencing LC‐MS/MS	No	SLE renal flare prediction
		Alaiya *et al*. 32	Renal Biopsies	6 LN class IV‐Global 5 LN class IV‐Segmental /3 ANCA‐GN, 3 normal kidneys	2‐DE, MALDI‐TOF/MS	Yes	LN diagnosis
4	Serotransferrin	Alaiya *et al*. 32	Renal Biopsies	6 LN class IV‐Global 5 LN class IV‐Segmental /3 ANCA‐GN, 3 normal kidneys	2‐DE, MALDI‐TOF MS	Yes	LN diagnosis
		Somparn *et al*. 30	Urine	5 active LN/ 5 age‐ and sex‐matched inactive LN	2‐DE, ESI‐Q‐TOF MS/MS	No	LN activity and diagnosis
5	Ezrin	Caster *et al*. 11	Serum (autoantibodies)	10 PLN/ 15 MLN, 10 non‐LN SLE	SDS‐PAGE, LC‐MS/MS	Yes	Diagnosis of proliferative forms of LN
		Alaiya *et al*. 32	Renal Biopsies	6 LN class IV‐Global 5 LN class IV‐Segmental /3 ANCA‐GN, 3 normal kidneys	2‐DE, MALDI‐TOF MS	Yes	LN diagnosis
6	Elongation factor 1‐alpha 1	Caster *et al*. 11	Serum (autoantibodies)	10 PLN/ 15 MLN, 10 non‐LN –SLE	SDS‐PAGE, LC‐MS/MS	Yes	Diagnosis of proliferative forms of LN
		Zhou *et al*. 24	PBMCs	14 SLE/ 9 age‐ and sex‐matched HC	2‐DE, LC‐MS/MS	Yes	SLE‐related thrombophilia (increased intracellular and decreased serum annexin A5 levels are protective)
7	Glyceraldehyde‐3‐phosphate dehydrogenase (G3PD)	Caster *et al*. 11	Serum (autoantibodies)	10 PLN/ 15 MLN, 10 non‐LN SLE	SDS‐PAGE, LC‐MS/MS	Yes	Diagnosis of proliferative forms of LN
		Serada *et al*. 17	Serum (autoantibodies)	41 SLE (23 LN)/ 60 DC, 19HC	2D‐PAGE, WB, LC‐MS/MS ELISA	No	LN Diagnosis
		Zhou *et al*. 24	PBMCs	14 SLE/ 9 age‐ and sex‐matched HC	2‐DE, LC‐MS/MS	Yes	SLE‐related thrombophilia (increased intracellular and decreased serum AnxA5 levels are protective)
8	Alpha‐enolase	Caster *et al*. 11	Serum (autoantibodies)	10 PLN/ 15 MLN, 10 non‐LN SLE	SDS‐PAGE, LC‐MS/MS	Yes	Diagnosis of proliferative forms of LN
		Zhou *et al*. 24	PBMCs	14 SLE/ 9 age‐ and sex‐matched HC	2‐DE, LC‐MS/MS	Yes	SLE‐related thrombophilia (increased intracellular and decreased serum annexin A5 levels are protective)
9	Haptoglobin	Somparn *et al*. 30	Urine	5 active LN/ 5 age‐ and sex‐matched inactive LN	2‐DE, ESI‐Q‐TOF MS/MS	No	LN activity and diagnosis
		Kazemipour *et al*. 14	Serum	13 SLE/ 7 HC	2‐DE, MALDI‐TOF/ TOF MS	No	SLE diagnosis
10	Transthyretin	Somparn *et al*. 30	Urine	5 active LN/ 5 age‐ and sex‐matched inactive LN	2‐DE, ESI‐Q‐TOF MS/MS	No	LN activity and diagnosis
		Kazemipour *et al*. 14	Serum	13 SLE/ 7 HC	2‐DE, MALDI‐TOF/ TOF MS	No	SLE diagnosis
11	Apolipoprotein A‐I	Kazemipour *et al*. 14	Serum	13 SLE/ 7 HC	2‐DE, MALDI‐TOF/ TOF MS	No	SLE diagnosis
	Apolipoprotein CIII	Morgan *et al*. 16	Serum	7 SLE/ 7 age‐, sex‐ and race‐matched HC	2‐DE, LC‐MS/MS	Yes	LN diagnosis Increased atherosclerotic risk in LN
	Apolipoprotein C‐I	Wang *et al*. 23	PBMCs	6 active SLE, 6 stable SLE/6 Rheumatoid Arthritis, 6 age‐ and sex‐matched HC	MALDI‐TOF/TOF MS iTRAQ‐labelled	No	SLE diagnosis and activity
	Apolipoprotein A‐IV precursor	Dai *et al*. 19	PBMCs	9 SLE/ 7 age‐matched HC	2‐DE, MALDI‐TOF/TOF MS	No	SLE diagnosis

PLN: proliferative lupus nephritis; MLN: membranous lupus nephritis; LN: lupus nephritis; SLE: systemic lupus erythematosus; SDS‐PAGE: sodium dodecyl sulphate–polyacrylamide gel electrophoresis; LC: liquid chromatography; MS: mass spectrometry; DC: disease controls; HC: healthy controls; 2D‐PAGE: two‐dimensional polyacrylamide gel electrophoresis; WB: Western blot analysis; ELISA: enzyme‐linked immunosorbent assay; iTRAQ: isobaric tagging reagent for absolute quantitation; 2‐DE: two‐dimensional gel electrophoresis; MALDI: matrix‐assisted laser desorption/ionization; TOF: time‐of‐flight mass spectrometry; ANCA: antineutrophil cytoplasmic antibody vasculitis; GN: glomerulonephritis; PBMCs: peripheral blood mononuclear cells; ESI‐Q: electrospray ionization quadrupole; SELDI: surface‐enhanced laser desorption/ionization.

## Discussion

The aim of this systematic review was to critically review proteomic biomarkers identified in patients with SLE using MS‐based proteomics. Although in recent years many SLE protein biomarker reports have been published, this is the first attempt to present a systematic review on this important topic. This review summarizes the candidate proteomic biomarkers identified so far in SLE, as well as their potential disease association and clinical use.

This review has revealed 241 potential SLE protein biomarkers that have been identified by MS‐based proteomics, using both targeted and untargeted approaches. Due to the large number of proteins detected, herein we are discussing the most promising biomarkers for SLE by focusing on two particular aspects. The first aspect includes studies in which proteins were validated in an independent cohort, using either MS or immunobased techniques, and the second includes the studies that had identified common biomarkers. In order to present a more targeted discussion, we divided the validated protein biomarkers into three main categories, based on their association with different clinical aspects of the disease. These categories are as follows: (*i*) SLE biomarkers, (*ii*) LN biomarkers and (*iii*) biomarkers associated with neuropsychiatric SLE (NPSLE).

### SLE biomarkers

The first category includes the most promising biomarkers that are suitable for SLE diagnosis or activity assessment. An increased expression of phosphorylated S100A9 isoforms was detected in the proteome of SLE PBMCs, suggesting abnormal S100A9 signalling, as well as reflecting the increased numbers of circulating low‐density granulocytes in these patients 21. S100A9 is a pro‐inflammatory protein, expressed mainly in the cytosol of neutrophils and monocytes 43, and it was suggested that mature neutrophils recruited to the inflammatory sites may result in local S100A9 release, which induces neutrophil degranulation 44. These activated neutrophils contribute to SLE pathogenesis *via* many mechanisms, including their ability to form neutrophil extracellular traps (NETs), and produce increased interferon‐α 45, 46.

Serine‐threonine kinase receptor‐associated protein is another important biomarker that was found to be under‐expressed in PBMCs of active SLE 22. STRAP was inversely correlated to *SLEDAI,* suggesting an association with a favourable clinical course in patients with SLE and could thus be used as a potential biomarker of clinical SLE activity/severity. The mechanisms of action of STRAP in SLE may be attributed to its interaction, either with transforming growth factor‐β receptor 47 or with apoptosis signal‐regulating kinase 1 48.

### LN biomarkers

A significant number of biomarkers were found to be associated with LN diagnosis or clinical activity/severity. Five biomarkers for diagnosing LN in kidney biopsies of class IV LN patients including serotransferrin, cytokeratin 18, cytokeratin 19, albumin and annexin A5 were identified 32.

Serotransferrin, an iron‐binding transport protein coregulated by interferon‐α, plays a role in iron metabolism and the innate immune system 49, 50. Plasma levels of serotransferrin were associated with SLE disease activity 49 and high urinary levels were associated with paediatric LN activity and severity, suggesting that it may be used as a predictive LN biomarker 51. More recently, transferrin in combination with other urine biomarkers predicted the decline of renal function in LN patients 52.

Cytokeratins (CKs) are a family of intermediate filament proteins, comprising 20 known CKs, which are classified into type I keratins (K9−K20) or type II keratins (K1–K8) 53. CKs, particularly cytokeratins 18 and 19, undergo caspase‐mediated degradation during apoptosis, in which organized cell fragmentation prevents initiation of inflammatory responses. Thus, programmed destruction of cytokeratin components may affect the sensitivity of the cell to apoptose 54, 55, 56. CKs have also been associated with other autoimmune diseases. Particularly, increased levels of anti‐CK18 and anti‐CK19 antibodies were observed in the sera of patients with autoimmune hepatitis 57. In the kidney, they were recently shown to represent early markers of tubular injury and stress 58.

Annexins are calcium‐dependent, phospholipid‐binding proteins 59, involved in several cell functions including vesicle trafficking, calcium signalling, cell growth, division and apoptosis 60. Some of the annexins have anti‐inflammatory actions. Annexins A1 and A2 play a crucial role in the phagocytosis of apoptotic lymphocytes, reducing inflammation through the release of immunosuppressive cytokines 61. Annexins could be used as potential LN biomarkers; however, their implication in LN pathogenesis is not known. Annexin A5 was found to be elevated in kidney biopsy samples of class IV, LN patients 32 and was associated with SLE‐related thrombophilia 24. Heterogeneous transcellular distribution of annexin A5 in patients with SLE, which is increased in PBMCs and decreased in sera, indicated a protective response to SLE‐related thrombophilia 24. Annexins A2 and A5 have a high affinity for phospholipids, which are involved in the regulation of the coagulation cascade. Antibodies against annexins A5 and A2 were detected in thrombotic‐associated diseases and other autoimmune diseases besides SLE, such as primary antiphospholipid syndrome and systemic sclerosis 60. Besides annexin A5, anti‐annexin A2 antibodies were also reported as possible biomarkers for the proliferative form of LN (PLN, class III or IV). Serum anti‐annexin A2 antibody levels discriminated PLN patients, not only from patients with other autoimmune diseases and healthy controls, but also from patients with a membranous form of LN 11. Annexin A2 was also identified as a target of serum antibodies in patients with SLE 17. In addition, studies in LN patients and SLE‐prone mice showed that annexin A2 facilitates the binding of anti‐dsDNA antibodies to mesangial cells, contributing to the LN pathogenesis 62.

Serum apolipoprotein CIII (apoCIII) was associated with an increased atherosclerotic risk in LN patients 16. Apo‐CIII involved in the regulation of triglyceride level and elevated apo‐CIII production is related with hypertriglyceridemia 63. In this content, increased apo C‐III levels in patients with SLE may result from increased plasma very low‐density lipoprotein (VLDL) cholesterol and triglycerides and decreased high‐density lipoprotein (HDL) cholesterol 16. Finally, Apo C‐III‐containing Apo B lipoprotein subclasses were associated with increased atherosclerosis risk, in patients with rheumatoid arthritis 64.

Urinary prostaglandin H_2_D‐isomerase (PGDS) was identified as a candidate biomarker for LN activity 30. PGDS catalyses the conversion of prostaglandin H_2_ (PGH2) to PGD2, which is implicated in physiological processes such as sleep regulation, prevention of platelet aggregation, allergy and inflammation 65, 66. Studies on monkeys demonstrated *de novo* synthesis of PGDS in podocytes, and Bowman's capsule of the glomeruli 67. Interestingly, in paediatric SLE patients urinary PGDS was associated with LN activity. However, no comparison with non‐LN glomerular diseases was performed 51. In murine SLE models, elevated levels of urinary PGDS were associated with LN severity 68 and PGDS correlated with glomerular inflammation in adriamycin‐induced nephropathy in mice 69.

Hepcidin is a 25‐amino acid peptide hormone mainly produced by hepatocytes and a key regulator of systemic iron homeostasis 70, 71. It is known to be involved in the pathogenesis of the anaemia of chronic inflammation including that of chronic kidney disease 71. It appears to be associated with proinflammatory cytokines such as interleukin‐6 and tumour necrosis factor‐α 72, 73, molecules that are known to be implicated in SLE pathogenesis 74, 75. Hepcidin isoforms were differentially expressed in the urine of LN patients during renal flares, indicating hepcidin as a promising biomarker for the assessment of renal severity in LN patients. Interestingly, it was suggested that hepcidin may be produced within kidney during renal flare, rather than being only filtered 31. Additionally, serum pro‐hepcidin levels were shown to reflect disease activity, in patients with rheumatoid arthritis 76.

### Biomarkers associated with NPSLE

SLE affects both the central and the peripheral nervous system. NPSLE is a common manifestation of SLE, with a prevalence of 14–80% in adults and 22–95% in children. It has been suggested that NPSLE pathogenesis may be associated with autoantibody‐mediated neuronal dysfunction and vasculopathy 77.

Anti‐αGDI antibody was identified as a potential diagnostic biomarker of psychosis‐associated NPSLE 15. αGDI is a small GTP‐binding protein, which is involved in the regulation of vesicle trafficking 78. In addition, it is a brain‐specific antigen, which is localized in neurons 79, 80. Mutations in the *Gdi1* gene that encodes αGDI were reported in families with X‐linked non‐specific mental retardation 81. Experiments in *Gdi1‐*deficient mice demonstrated damage of associative memory as well as changes in social behaviour without any anatomic abnormality 82. Kimura and colleagues suggested that the function of αGDI may be prevented by anti‐αGDI antibodies, affecting the exocytosis of synaptic vesicles during neurotransmitter release that is related with psychosis in patients with NPSLE 15. More recent studies in mice suggest a key role of aGDI by specific Ras‐related protein in brain (RAB) GTPases acting specifically in forebrain regions at the pre‐synaptic sites involved in memory formation 83.

Ubiquitin is a small regulatory protein, member of a family of structurally conserved proteins, which regulate numerous processes in eukaryotic cells. The most significant function of ubiquitin is targeting proteins for degradation 84. Ubiquitin is part of the ubiquitin‐proteasome system, which is involved in several important cellular processes such as regulation of apoptosis, cell cycle progression, cell division, cell development and differentiation, cell trafficking as well as modulation of immune and inflammatory responses 85. Imbalances in the ubiquitin‐proteasome system may lead to systemic autoimmunity and neurodegenerative disorders 86 and elevated levels of ubiquitin in cerebrospinal fluid (CSF) of NPSLE are associated with disease activity 36. Studies in other neurological disorders, including Creutzfeldt–Jakob 87 and Alzheimer's disease 88, demonstrated also elevated ubiquitin levels in the CSF of patients, indicating a possible role of ubiquitin in neural degradation and apoptosis.

### Biomarkers identified in multiple studies

This systematic review has also identified 11 biomarkers that were detected in at least two independent studies (Table 3). These biomarkers are as follows: annexins A2 and A5, alpha‐1‐antitrypsin (A1AT), serotransferrin, ezrin, elongation factor‐1‐alpha 1, glyceraldehyde‐3‐phosphate dehydrogenase, alpha‐enolase, haptoglobin, transthyretin and apolipoproteins (A‐I, CIII, C‐I, A‐IV) (Table S1). Four of these, namely annexins A2 and A5, serotransferrin and apolipoproteins, were also validated in independent cohorts and were discussed above. It is noteworthy that 5 of the 11, annexins A2 and A5, A1AT, serotransferrin and ezrin were detected in kidney biopsies and it is of interest that these were also present in serum, urine and/or PBMCs, supporting the notion that these biomarkers can be utilized as potential LN diagnostic tools.

In this content, A1AT was found in the urine of active LN and during SLE renal flares 31, as well as in renal tissue of class IV LN patients 32. A1AT is an acute phase protein and the most prominent circulating protease inhibitor that also plays an important role in regulating immunity, inflammation and apoptosis 89. Although it is mainly produced in the liver, it was shown that A1AT is also produced in the kidney in response to injury 90 and confers cytoprotective effects 90, 91. Moreover, it was found in the urine and serum of patients with GN 92 and in the urine of patients with SLE, distinguishing them from patients suffering from other proteinuric diseases 93.

Ezrin was identified as a candidate antigen for serum autoantibodies in proliferative LN forms 11; it was also found to be upregulated in class IV nephritis kidney tissue 32. Ezrin is the prototypic member of the ezrin protein subfamily, which serves as a linker between the plasma membrane and the cytoskeleton. Indeed, these proteins act as intracellular scaffolds 94, 95, regulating B‐ and T‐cell activation 96. A recent study showed that ezrin plays a role in regulating inflammation, *via* limiting the B‐cell IL‐10 production 97.

Elongation factor 1‐alpha 1 (EF‐1A1) was also a candidate antigen in proliferative LN and was downregulated in PBMCs from patients with SLE, compared with healthy controls 24. Moreover, elongation factor 1‐gamma (EF‐1γ) was identified as a target antigen of serum antibodies in patients with SLE compared with RA, polymyositis and healthy controls 17. Elongation factor 1 (EF1) is a major translational factor that consists of four different subunits, EF‐1αβγδ. Apart of its canonical function, EF‐1 is a multifunctional protein that has been implicated in various important cellular processes such as cell growth, signal transduction, cytoskeletal organization apoptosis and tumorigenesis 98.

Another interesting protein, glyceraldehyde‐3‐phosphate dehydrogenase (GAPDH), was found to be a candidate antigen for antibodies in patients with proliferative LN 11 and in patients with SLE compared with RA, polymyositis and healthy controls 17. GAPDH was also downregulated in PBMCs from patients with SLE compared with healthy controls 24. GAPDH is a classical glycolytic enzyme involved in energy production, but it is also implicated in numerous important cellular pathways including receptor‐mediated cell signalling, transcriptional and post‐transcriptional gene regulation, maintenance of DNA integrity, oxidative stress response and apoptosis 99. In patients with SLE, GAPDH was found to interact with proliferating cell nuclear antigen, a known autoantigen targeted by antibodies, indicating its possible role in autoimmune responses induction against proliferating cell nuclear antigen complexes in SLE 100.

In addition, alpha‐enolase was identified as a candidate antigen in proliferative LN forms 11 and was upregulated in SLE PBMCs compared with healthy controls 24. Alpha‐enolase is a multifunctional glycolytic enzyme that is involved in various biological and pathophysiological processes 101, 102. Evidence revealed that α‐enolase plays a role in systemic autoimmune diseases. Anti‐α‐enolase antibodies were present in patients with SLE with active LN and other autoimmune diseases such as systemic sclerosis 103. In addition, anti‐α‐enolase IgG2 levels were increased in LN serum, enabling the discrimination between SLE patients with LN, SLE patients without LN and patients with rheumatoid arthritis 104.

Transthyretin and haptoglobin (Hp) were upregulated in urine samples from active LN 30 patients, compared with inactive LN and were also be upregulated in the serum proteome of patients with SLE, compared to healthy controls 14. Transthyretin is a serum and cerebrospinal fluid transporter of the thyroid hormone thyroxine (T4) and retinol 105. It has been associated with numerous disorders including, familial amyloid cardiomyopathy and senile systemic amyloidosis 106, 107. A pilot study showed that transthyretin levels were upregulated in the sera of paediatric SLE patients compared to healthy controls 108. Additionally, increased serum levels of transthyretin were associated with the severity of rheumatoid arthritis, suggesting a role in disease pathogenesis 109. The main biological role of Hp is to bind haemoglobin, prevent iron loss and subsequent kidney damage during haemolysis. It is also an acute phase protein with antioxidant and immunomodulatory properties. Hp expression was associated with inflammatory autoimmune diseases, including arthritis and SLE, and as a marker of disease activity 110. Plasma levels of Hp were associated with disease severity in patients with SLE 111. In addition, Hp2‐2 phenotype was found to be over‐represented in patients with SLE, and may be contribute to cardiovascular complications in SLE due to its lower antioxidant capacity 111, 112.

### Genome research of lupus

Finally, advances in genomic technologies during the past decade have enabled the identification of numerous risk genetic factors associated with susceptibility to SLE. To date, more than 60 SLE susceptibility loci have been identified by genome‐wide association studies (GWAS) in different population cohorts, including *HLA, STAT4* and *IRF5*. However, risk variants identified so far explain only a small fraction of the overall SLE heritability 113. A number of next‐generation DNA sequencing (NGS) methodologies have been used not only to validate previously identified susceptibility genes and loci associated with SLE such as *IRF2, IRF5, UBE2L3, IFIHI, TNIP1, TNFAIP3* and *BLK*, but also enabled the discovery of additional gene variants, especially rare variants that are not identified by GWAS 114, 115. Although the genetic aspects of SLE are beyond the scope of this review, we attempted to correlate the protein biomarkers summarized in this review with susceptibility genes identified using high‐throughput genomic methodologies such as NGS and GWAS 113, 114, 115, 116. Overall, no direct associations were found linking proteins or biomarkers as described in this review to their corresponding genes or loci. This may be partly explained by the fact that most of GWAS identified susceptibility loci are located within non‐coding DNA regions. Evidently these have no apparent role in encoding proteins, suggesting a possible regulatory role of these variants in protein dysfunction and consequently in disease pathology 117. In addition, epigenomics may explain part of the missing heritability 118. Recently, the term ‘proteogenomics’ has been introduced, which is an area of research at the interface of genomics and proteomics, with great potential towards the discovery of biomarkers for many diseases and in particular SLE 119, 120.

### Study limitations

An ideal SLE biomarker would be biologically and pathophysiologically relevant, reproducible, simple to apply in routine practice (inexpensive, easy and rapid to quantify) and would have a high degree of sensitivity and specificity 121. At present, no SLE biomarker exists that fulfils all of the above. It is appreciated that some of the above‐described candidate biomarkers are non‐specific stress proteins, linked with a multitude diseases and conditions. One of the main limitations of the majority of the reviewed studies was the small sample size and lack of disease control groups. Other limitations that emerged include the absence of details about the ethnic group and age of the subjects as well as lack of validation of the results. In addition, although a number of studies validated their results in an independent cohort, the size of the cohort used for the validation was not always sufficient. Finally, bias due to the absence of standardized protocol for preparing and presenting patient samples constitutes another important limitation, showed by most of the reviewed studies.

### Future prospects

Although a significant number of SLE biomarker reports have been published to date, there are still many challenges that need to be overcome in future proteomic studies that aim to identify clinically useful SLE biomarkers. Firstly, differences in proteomic results across different studies can be attributed to differences in the selection criteria of the samples. A recent published study uncovered the molecular heterogeneity of SLE, providing an explanation for the failure of the clinical trials 122. Thus, there is a need to establish universally accepted sample selection criteria in order to better streamline phenotype–genotype correlations and make results across different proteomic studies more comparable. Future studies need to recruit a larger number of patients. Secondly, MS identified SLE biomarkers should be validated in multicentre studies using standardized immunobased proteomic techniques or other MS methodologies. In addition, future proteomic studies should focus on biomarkers that have already been identified in multiple studies and in several, invasive as well as non‐invasive specimen types. Due to the heterogeneous nature of SLE, it is more likely that a panel of proteomic biomarkers rather than a single protein will be needed by the physicians, for SLE diagnosis and treatment. Therefore, studies working with panels of biomarkers that are involved in biologically relevant pathways may be more meaningful and more substantial than studies focus on single biomarkers. Finally, although promising panels of biomarker protein peaks have been identified, the exact identity of the detected proteins is still not known.

## Conclusions

Advances in high‐throughput MS technologies have undoubtedly created new avenues for discovering sensitive and specific SLE biomarkers. MS‐based proteomics have been used to study a plethora of biological specimens from patients with SLE, leading to the identification of biomarkers related to disease diagnosis and activity as well as to specific organ involvement. There are already a number of valuable MS‐based proteomic studies in the literature that fulfil most of the requirements for clinical proteomic biomarker reporting. A significant number of potential biomarkers have been identified to be associated with many clinical aspects of SLE, including diagnosis, disease activity and prognosis. It is noteworthy that almost half of these studies have validated their results in an independent cohort. What is lacking and should be addressed in future biomarker studies, is the use of larger patient cohorts, as well as the validation of already identified biomarkers in independent patient cohorts. Furthermore, protein biomarkers, which have been already identified in multiple studies, particularly those that were also detected in non‐invasive biological samples, hold a great promise. Such biomarkers should form the backbone and be at the forefront of executing larger multicentre studies in future using well‐characterized patient cohorts, in order to prove their clinical utility.

## Conflict of interest

The authors declare that they have no competing interests.

## Author contributions

The manuscript was written through contributions of all authors. All authors have given approval to the final version of the manuscript.

## Supporting information


**Figure S1** Flow diagram showing the systematic literature search and review process. All methods were applied in accordance with the PRISMA guidelines.
**Table S1.** Requirements for scientific reporting of proteomic biomarker data reported by Mischak *et al*. 2010 implemented for the 25 articles obtained in the systematic review.
