# Supplementary material for: Biomarkers of systemic lupus erythematosus identified using mass spectrometry‐based proteomics: a systematic review
Source: J Cell Mol Med. 2016 Nov 23;21(5):993–1012. doi: 10.1111/jcmm.13031 (PMC5387176; doi:10.1111/jcmm.13031)
Supplement: Supplementary file 1 — Figure S1 Flow diagram showing the systematic literature search and review process. All methods were applied in accordance with the PRISMA guidelines. Table S1. Requirements for scientific reporting of proteomic biomarker data reported by Mischak et al. 2010 implemented for the 25 articles obtained in the systematic review. [file JCMM-21-993-s001.docx]

**Supporting Information**

**Figure S1:** Flow diagram showing the systematic literature search and review process. All methods were applied in accordance with the PRISMA guidelines.

**Table S1.** Requirements for scientific reporting of proteomic biomarker data reported by Mischak et al. 2010 implemented for the 25 articles obtained in the systematic review.

**Figure S1.**

Records identified via MEDLINE (n=396)

Total records after duplicates removing (n=775)

Records identified through EMBASE (n=697)

Records screened (n=775)

Exclusion of review articles, editorials, case reports, letters to the editor, conference abstracts, notes, news

(n=327)

Total number of studies included in the systematic review (n=25)

Full-text articles excluded because they did not meet the eligibility criteria:

Articles not in English (n=12)

Studies not on Human (n=36) Studies did not refer to SLE (n=230) Studies did not use MS (n=45)

Studies not related to biomarker discovery (n=70)

Drug related studies (n=26)

Pediatric/Juveline SLE (n=4)

Juvenile SLE (n=1)

Full-text articles assessed for eligibility (n=448)

**Table S1.**

| Requirements for scientific reporting | Articles that fulfil the requirements |
| --- | --- |
| Justification and description of the clinical question, outcomes, and selection of subjects | All articles: Alaiya A. et al. (2015); Caster D. J. et al. (2015); Dai, Y. et al. (2008); Fang, S. et al. (2008); Huang, Z. et al. (2009); Iizuka, N. et al. (2009); Kazemipour, N. et al. (2015); Kimura, A. et al. (2010); Li, Y. et al. (2013); Morgan, P.E. et al. (2007); Mosley, K. et al. (2006); Nielsen, C. T. (2012); Nielsen, C. T. (2015); Ostergaard, O. et al. (2013); Pavon, E. J. et al. (2012); Serada, S. et al. (2007); Somparn, P. et al. (2012); Sui, W. et al. (2012); Sun, L. et al. (2011); Wang, L. et al. (2011); Wang, L. et al. (2012); Wu, Y. et al. (2009); Zhang, X. et al. (2008); Zhou, D. et al. (2012); Zhou, X.S. et al. (2013); |
| Subjects description assessment | Huang, Z. et al. (2009) *; Iizuka, N. et al. (2009)*; Kazemipour, N. et al. (2015); Morgan, P.E. et al. (2007); Mosley, K. et al. (2006)*; Nielsen, C. T. (2012); Nielsen, C. T. (2015); Ostergaard, O. et al. (2013)*; Somparn, P. et al. (2012)*; Sun, L. et al. (2011)**; Wang, L. et al. (2011)*; Wang, L. et al. (2012)*; Wu, Y. et al. (2009); Zhou, D. et al. (2012); Zhou, X.S. et al. (2013)* |
| Description of samples | Alaiya A. et al. (2015); Caster D. J. et al. (2015); Dai, Y. et al. (2008); Fang, S. et al. (2008); Iizuka, N. et al. (2009); Kazemipour, N. et al. (2015); Kimura, A. et al. (2010); Li, Y. et al. (2013); Morgan, P.E. et al. (2007); Mosley, K. et al. (2006); Nielsen, C. T. (2012); Nielsen, C. T. (2015); Ostergaard, O. et al. (2013); Pavon, E. J. et al. (2012); Serada, S. et al. (2007); Somparn, P. et al. (2012); Sui, W. et al. (2012); Sun, L. et al. (2011); Wang, L. et al. (2011); Wang, L. et al. (2012); Zhang, X. et al. (2008); Zhou, D. et al. (2012); Zhou, X.S. et al. (2013); |
| Description of experimental methodology | Alaiya A. et al. (2015); Caster D. J. et al. (2015); Dai, Y. et al. (2008); Fang, S. et al. (2008); Iizuka, N. et al. (2009); Kazemipour, N. et al. (2015); Kimura, A. et al. (2010); Li, Y. et al. (2013); Morgan, P.E. et al. (2007); Mosley, K. et al. (2006); Nielsen, C. T. (2012); Nielsen, C. T. (2015); Ostergaard, O. et al. (2013); Pavon, E. J. et al. (2012); Serada, S. et al. (2007); Somparn, P. et al. (2012); Sui, W. et al. (2012); Sun, L. et al. (2011); Wang, L. et al. (2011); Wang, L. et al. (2012); Wu, Y. et al. (2009); Zhang, X. et al. (2008); Zhou, D. et al. (2012); Zhou, X.S. et al. (2013) |
| Description of the statistical evaluation | Alaiya A. et al. (2015); Caster D. J. et al. (2015); Dai, Y. et al. (2008); Fang, S. et al. (2008); Huang, Z. et al. (2009); Iizuka, N. et al. (2009); Kazemipour, N. et al. (2015); Kimura, A. et al. (2010); Li, Y. et al. (2013); Morgan, P.E. et al. (2007); Mosley, K. et al. (2006); Nielsen, C. T. (2012); Nielsen, C. T. (2015); Ostergaard, O. et al. (2013); Pavon, E. J. et al. (2012); Somparn, P. et al. (2012); Sun, L. et al. (2011); Wang, L. et al. (2011); Wang, L. et al. (2012); Wu, Y. et al. (2009); Zhang, X. et al. (2008); Zhou, D. et al. (2012); Zhou, X.S. et al. (2013); |
| Validation of the results | Alaiya A. et al. (2015); Caster D. J. et al. (2015); Huang, Z. et al. (2009); Kimura, A. et al. (2010); Morgan, P.E. et al. (2007); Mosley, K. et al. (2006); Nielsen, C. T. (2015); Pavon, E. J. et al. (2012); Somparn, P. et al. (2012); Sun, L. et al. (2011); Wang, L. et al. (2011); Zhang, X. et al. (2008); Zhou, D. et al. (2012); |
| Limitation acknowledgment | Alaiya A. et al. (2015); Fang, S. et al. (2008); Kimura, A. et al. (2010); Morgan, P.E. et al. (2007); Mosley, K. et al. (2006); Nielsen, C. T. (2012); Nielsen, C. T. (2015); Ostergaard, O. et al. (2013); Zhang, X. et al. (2008); Zhou, D. et al. (2012) |
| Author contribution statement | Nielsen, C. T. (2012); Ostergaard, O. et al. (2013) |
